# Grain‐sized moxibustion promotes NK cell antitumour immunity by inhibiting adrenergic signalling in non–small cell lung cancer

**DOI:** 10.1111/jcmm.16320

**Published:** 2021-01-27

**Authors:** Dan Hu, Weiming Shen, Chenyuan Gong, Cheng Fang, Chao Yao, Xiaowen Zhu, Lixin Wang, Chen Zhao, Shiguo Zhu

**Affiliations:** ^1^ School of Acupuncture, Moxibustion and Tuina Shanghai University of Traditional Chinese Medicine Shanghai China; ^2^ Center for Traditional Chinese Medicine and Immunology Research School of Basic Medical Sciences Shanghai University of Traditional Chinese Medicine Shanghai China; ^3^ Department of Immunology and Pathogenic Biology School of Basic Medical Sciences Shanghai University of Traditional Chinese Medicine Shanghai China

**Keywords:** adrenergic signalling, antitumour immunity, moxibustion, NK cells, non–small cell lung cancer

## Abstract

Lung cancer is the leading cause of cancer‐related death worldwide, and non–small cell lung cancer (NSCLC) accounts for 85% of lung cancer diagnoses. As an ancient therapy, moxibustion has been used to treat cancer‐related symptoms in clinical practice. However, its antitumour effect on NSCLC remains largely unexplored. In the present study, a Lewis lung cancer (LLC) xenograft tumour model was established, and grain‐sized moxibustion (gMoxi) was performed at the acupoint of Zusanli (ST36). Flow cytometry and RNA sequencing (RNA‐Seq) were used to access the immune cell phenotype, cytotoxicity and gene expression. PK136, propranolol and epinephrine were used for natural killer (NK) cell depletion, β‐adrenoceptor blockade and activation, respectively. Results showed that gMoxi significantly inhibited LLC tumour growth. Moreover, gMoxi significantly increased the proportion, infiltration and activation of NK cells, whereas it did not affect CD4^+^ and CD8^+^ T cells. NK cell depletion reversed gMoxi‐mediated tumour regression. LLC tumour RNA‐Seq indicated that these effects might be related to the inhibition of adrenergic signalling. Surely, β‐blocker propranolol clearly inhibited LLC tumour growth and promoted NK cells, and gMoxi no longer increased tumour regression and promoted NK cells after propranolol treatment. Epinephrine could inhibit NK cell activity, and gMoxi significantly inhibited tumour growth and promoted NK cells after epinephrine treatment. These results demonstrated that gMoxi could promote NK cell antitumour immunity by inhibiting adrenergic signalling, suggesting that gMoxi could be used as a promising therapeutic regimen for the treatment of NSCLC, and it had a great potential in NK cell–based cancer immunotherapy.

## 1 INTRODUCTION

Although the cancer‐related death rate is reduced with the development of novel treatments, the mortality of lung cancer death remains high in both males and females worldwide.^1^ Non–small cell lung cancer (NSCLC) accounts for 85% of all lung cancer diagnoses, and its 5‐year survival rate is still lower than 15%.^2^ Therefore, it is urgently necessary to develop new methods for the treatment of NSCLC.

As an ancient therapy, moxibustion has been long used to treat many different diseases in Asia, and now, it has been rapidly spread and used in clinic and family health care all over the world. Moxibustion is a type of heat therapy and elicits effect by a direct or indirect thermal stimulation at specific acupoints via burning moxa products. Moxibustion mainly includes mild moxibustion, grain‐sized moxibustion (gMoxi), ginger moxibustion and others. Among these, gMoxi is direct moxibustion at the acupoint and only produces very slight moxa smog.

Moxibustion has been shown to have a good effect on treating tumour‐related symptoms, such as fatigue,^3^, ^4^ pain,^5^, ^6^, ^7^ anorexia ^8^ and depression,^9^ and on alleviating surgery‐ and chemotherapy‐induced adverse effects.^10^ However, its antitumour effect on NSCLC remains largely unexplored. In the present study, we investigated the antitumour effect of gMoxi and its underlying mechanism using a mouse Lewis lung cancer (LLC) xenograft tumour model. We found that gMoxi significantly suppressed LLC tumour growth by inhibiting adrenergic signalling and then promoting natural killer (NK) cell antitumour immunity. This finding suggested that gMoxi could be used as a promising method for treatments of NSCLC, and it had a great potential in NK cell–based cancer immunotherapy.

## 2 MATERIALS AND METHODS

### 2.1 Reagents

FITC antimouse CD4 (100405), PE antimouse CD3 (100205), PerCP/Cy5.5 antimouse CD8 (100733) and APC antimouse NCR1/NKp46 (137608) antibodies were purchased from BioLegend Inc Propranolol hydrochloride (H32020133) was obtained from Jiangsu Yabang Aipusen Pharmaceutical Co., Ltd. Epinephrine hydrochloride (E4642) was provided by Sigma. Annexin V/Dead Cell Apoptosis Kit (556547) was supplied by BD Pharmingen™.

### 2.2 Cell lines and cell culture

Lewis lung cancer cells were obtained from the Cell Bank of the Chinese Academy of Sciences and maintained in high‐glucose DMEM (SH30243.01; Hyclone) supplemented with 10% foetal bovine serum (FBS; Biowest) and 1% penicillin‐streptomycin (60162ES76; YEASEN). YAC‐1 (ATCC^®^ TIB­160™) cells were cultured in RPMI‐1640 medium (SH30809.01B; Hyclone) supplemented with 10% FBS and 1% penicillin‐streptomycin. All cell lines were incubated at 37°C in a humidified atmosphere containing 5% CO2.

### 2.3 Subcutaneous xenograft tumour model and gMoxi

All animal‐related procedures were approved by the Institutional Animal Care and Use Committee at Shanghai University of Traditional Chinese Medicine. Male C57BL/6 mice (6 weeks of age) were purchased from Vital River and bred under an SPF environmental condition. A total of 5 × 10^5^ LLC cells were subcutaneously inoculated on the upper back of C57BL/6 mice on day 0. gMoxi was performed at the acupoint of Zusanli (ST36, locates 2 mm below and lateral to the anterior tubercle of the tibia of mice) with three or seven moxa cones at each acupoint every other day from day 1. For the sham control group, gMoxi was performed at the mouse tail. For NK cell depletion, antimouse NK1.1 antibody (PK136) (108702, BioLegend) was intraperitoneally injected (100 μg per mouse) on days 0, 7 and 14. For β‐adrenoceptor blockade, propranolol hydrochloride (Prop) was dissolved in the drinking water at a final concentration of 0.5g/L and given to the animals at 1 week before LLC inoculation. For β‐adrenoceptor activation, a total of 0.5 mg/kg epinephrine hydrochloride (Epi) was daily administered by intraperitoneal (IP) injection from day 1. Tumour sizes were monitored every 2 days from day 3 by using an electronic calliper, and tumour volumes were calculated using the following formula: V = (π/8)×a × b^2^, where V = tumour volume, a = maximum tumour diameter and b = minimum tumour diameter. After 3 weeks, mice were killed by using CO2, and tumours were excised, weighed and photographed.

### 2.4 Immunophenotype analysis

Tumours and spleens were isolated from the tumour‐bearing mice to detect immunophenotypes of T cells and NK cells. Tumours were cut into small pieces with scissors and digested with 0.1% collagenase (40507ES60; YEASEN) for 2 hours. Subsequently, tumour tissues and spleens were filtered through a 70‐μm filter to make single‐cell suspension and the red blood cell lysis buffer (420301; BioLegend) was used to remove erythrocytes. The cells were exposed to appropriate fluorescence‐conjugated antibodies at 4°C for 30 minutes in the dark, then washed and resuspended in PBS containing 1% FBS. Data were acquired by using a CytoFLEX LX (Beckman) and analysed by using FlowJo software.

### 2.5 Immunofluorescence staining

Fresh tumour tissues were excised from the tumour‐bearing mice treated with gMoxi or sham control and then embedded into frozen sections. The sections were blocked with 5% bovine serum albumin (BSA) for 30 minutes and sequentially incubated with antimouse NKp46 antibody (EPR23097‐35, ab233558; Abcam) at 4°C overnight and Cy3 AffiniPure Donkey Anti‐Rabbit lgG (H + L) (711‐165‐152; Jackson Immuno) for 30 minutes, followed by staining with 4′,6‐diamidino‐2‐phenylindole (DAPI; C1005, Beyotime) for 20 minutes. Images were captured using a laser scanning confocal microscope (ZEISS).

### 2.6 NK cell cytotoxicity assays

Target cell apoptosis was detected to access NK cell cytotoxicity by using Annexin V/Dead Cell Apoptosis Kit according to the manufacturer's instructions. Briefly, splenocytes and YAC‐1 cells were co‐incubated at an E: T ratio of 10:1 for 4 hours. The cells were then stained with Annexin V‐FITC and propidium iodide (PI) at room temperature for 15 minutes in the dark and analysed by flow cytometry.

### 2.7 RNA sequencing

RNA sequencing was performed to investigate the molecular signalling pathways of gMoix. Tumours were excised from the tumour‐bearing mice treated with gMoxi or sham control. Total RNA was isolated by RNeasy Mini Kit (74106; Qiagen) following the manufacturer's instructions. RNA‐Seq was performed by Shanghai Biotechnology Corporation using Illumina and then analysed using HISAT2 software. Genes with fold change ≥2 and *P* < .05 were identified as differentially expressed genes (DEGs). GO (Gene Ontology) and Kyoto Encyclopedia of Genes and Genomes (KEGG) enrichment analyses of DEGs were performed to better understand the biological functions of genes.

### 2.8 Statistical analysis

All data were expressed as mean ± standard error of means (SEM) and analysed by using SPSS statistical software. One‐way analysis of variance (ANOVA) and independent‐samples *t* test were used to assess statistical significance. Post hoc comparisons were made with the Newman‐Keuls multiple comparisons or Bonferroni's tests, where appropriate. A *P* value less than .05 was considered statistically significant.

## 3 RESULTS

### 3.1 gMoxi inhibits LLC tumour growth

**FIGURE 1:**
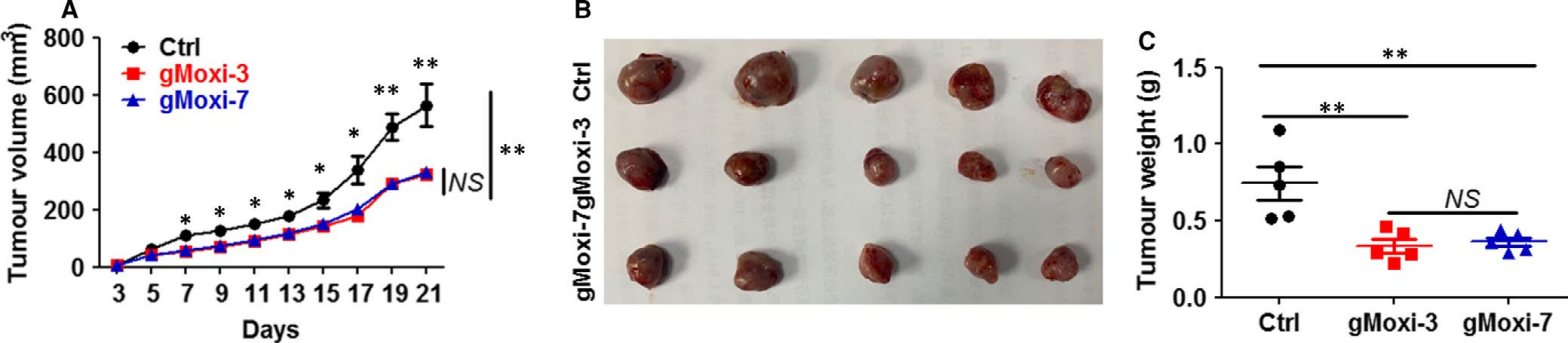
gMoxi suppresses Lewis lung cancer (LLC) tumour growth. LLC cells were inoculated on the upper back of C57BL6 mice on day 0, and gMoxi was performed at the acupoint Zusanli (ST36) with three or seven moxa cones every other day from day 1. Mice were killed on day 21, and tumours were excised and photographed. A, Tumour growth curve, and gMoxi‐3 and gMoxi‐7 represented gMoxi with three and seven moxa cones, respectively; B, tumour photograph; C, tumour weight. Independent experiments were repeated twice. **P < .01; NS, non‐statistically significant

To determine the antitumour effect of moxibustion on NSCLC, LLC cells were inoculated on the upper back of C57BL6 mice on day 0, and gMoxi was performed at Zusanli acupoint with three or seven moxa cones every 2 days from day 1. Results showed that gMoxi with either three or seven moxa cones significantly inhibited LLC tumour growth (Figure 1A). The tumours of mice treated with gMoxi were much smaller (Figure 1B) and lighter (Figure 1C) compared with those treated with sham gMoxi. Additionally, there was no clear difference in inhibitory effects between three and seven moxa cones. These results showed that gMoxi at Zusanli acupoint had the antitumour capacity without dose dependence.

### 3.2 gMoxi increases the proportion, infiltration and activation of NK cells

**FIGURE 2:**
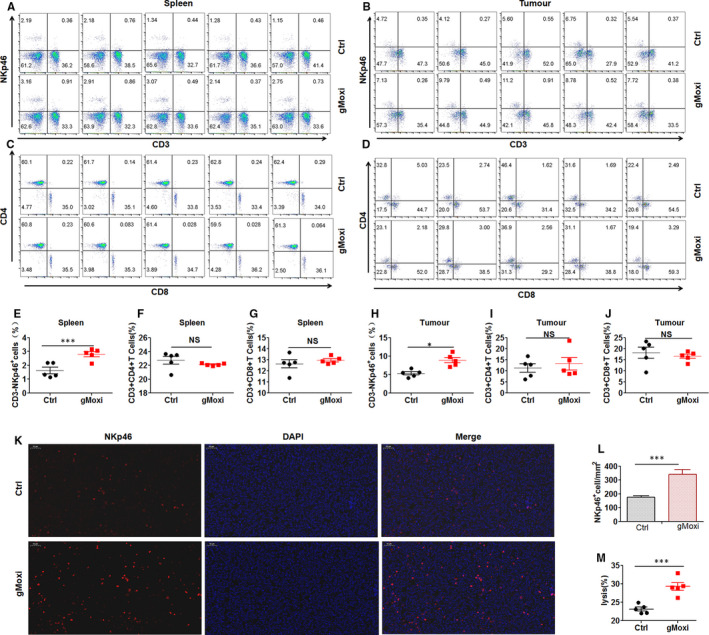
gMoxi increases NK cell antitumour immunity. Lewis lung cancer (LLC) cells were inoculated on the upper back of C57BL6 mice on day 0, and gMoxi was performed at the acupoint Zusanli (ST36) with three moxa cones every other day from day 1. Mice were killed on day 21, and tumours and spleens were isolated and analysed by flow cytometry or immunofluorescence staining. A, Spleen cells were stained with anti‐NKp46 and anti‐CD3; B, tumour cells were stained with anti‐NKp46 and anti‐CD3; C, spleen cells were stained with anti‐CD3, anti‐CD4 and anti‐CD8; D, tumour cells were stained with anti‐CD3, anti‐CD4 and anti‐CD8; E, the proportion of CD3^‐^NKp46^+^ NK cells in spleens; F, the proportion of CD3^+^CD4^+^ T cells in spleens; G, the proportion of CD3^+^CD8^+^ T cells in spleens; H, the proportion of CD3^‐^NKp46^+^ NK cells in tumours; I, the proportion of CD3^+^CD4^+^ T cells in tumours; J, the proportion of CD3^+^CD8^+^ T cells in tumours; K, frozen tumour sections were stained with anti‐NKp46 antibody and DAPI; L, the proportion of NKp46^+^ NK cells in tumours; M, splenocytes were co‐cultured with YAC1 cells at 10:1, and tumour lysis was detected by apoptosis analysis. Independent experiments were repeated twice. *P < .05; **P < .01; ***P < .001; NS, non‐statistically significant

As gMoxi inhibited LLC tumour growth, we next determined the underlying mechanism. NK cells, and CD4^+^ and CD8^+^ T cells play a crucial role in cancer immunosurveillance, and moxibustion has been shown to have an important regulatory effect on these cells.^11^ Therefore, the proportions of NK cells, and CD4^+^ and CD8^+^ T cells in spleens and tumours were analysed by flow cytometry (Figure S1 **)** or immunofluorescence staining. Results showed that gMoxi significantly increased the proportion of NK cells in spleens (Figure 2A,E) and infiltration in tumours (Figure 2B,H,K‐L), whereas it did not affect CD4^+^ and CD8^+^ T cells (Figure 2C‐D,F‐G,I‐J). Moreover, gMoxi dramatically enhanced splenocyte‐mediated cell killing (Figure 2M). As gMoxi clearly increased NK cells in spleens, NK cells kill target cells without the need of prior priming; therefore, the enhancement of splenocyte‐mediated cell killing by gMoxi should be due to NK cells, and this suggested that gMoxi could enhance NK cell–mediated cell killing. Taken together, these results indicated that gMoxi could promote NK cell antitumour immunity.

### 3.3 Tumour regression by gMoxi depends on NK cells

**FIGURE 3:**
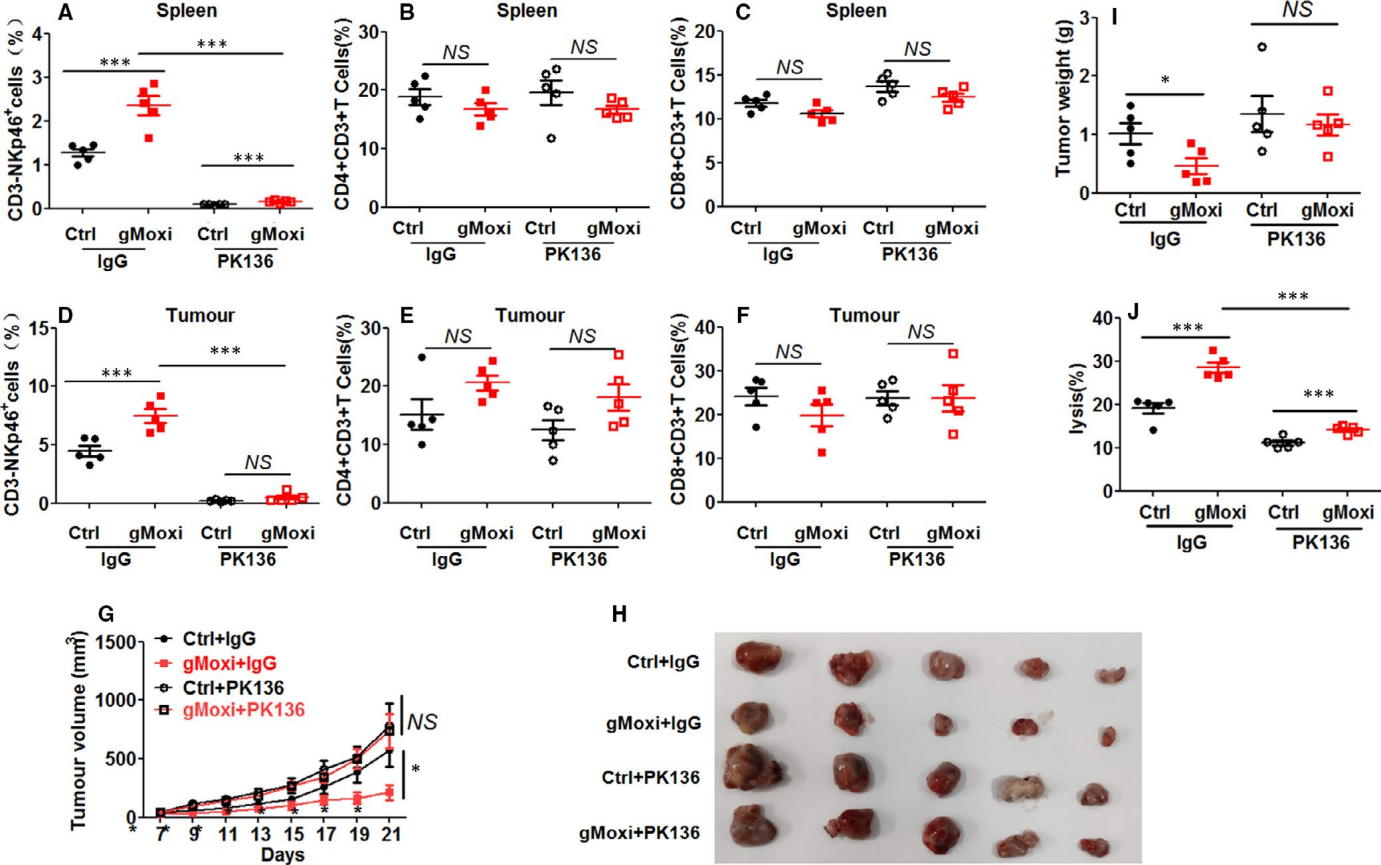
NK cell depletion reverses gMoxi‐mediated tumour regression. Lewis lung cancer (LLC) cells were inoculated on the upper back of C57BL6 mice on day 0, and gMoxi was performed at the acupoint Zusanli (ST36) with three moxa cones every other day from day 1. A total of 100 μg of PK136 per mouse was administered by IP injection on days 0, 7 and 14. Mice were killed on day 21, and tumours and spleens were isolated and analysed by flow cytometry. A, The proportion of CD3^‐^NKp46^+^ NK cells in spleens; B, the proportion of CD3^+^CD4^+^ T cells in spleens; C, the proportion of CD3^+^CD8^+^ T cells in spleens; D, the proportion of CD3^‐^NKp46^+^ NK cells in tumours; E, the proportion of CD3^+^CD4^+^ T cells in tumours; F, the proportion of CD3^+^CD8^+^ T cells in tumours; G, tumour growth curve; H, tumour photograph; I, tumour weight; J, splenocytes were co‐cultured with YAC1 cells at 10:1, and tumour lysis was detected by apoptosis analysis. Independent experiments were repeated twice. *P < .05; **P < .01; ***P < .001; NS, non‐statistically significant

As gMoxi could promote NK cell antitumour immunity, we next determined whether the tumour regression by gMoxi depended on NK cells. Therefore, NK cells were depleted by PK136 antibody. Results showed that NK cells in spleens (Figure 3A) and tumours (Figure 3D) were depleted by PK136 antibody, and CD4^+^ and CD8^+^ T cells in spleens (Figure 3B‐C) and tumours (Figure 3E‐F) were not significantly affected. NK cell depletion reversed the tumour regression (Figure 3G‐I) and NK cell–mediated cell killing (Figure 3J) by gMoxi. These results demonstrated that the tumour regression by gMoxi depended on NK cells.

### 3.4 The enhanced antitumour immunity of NK cells by gMoxi may be attributed to the inhibition of adrenergic signalling

**FIGURE 4:**
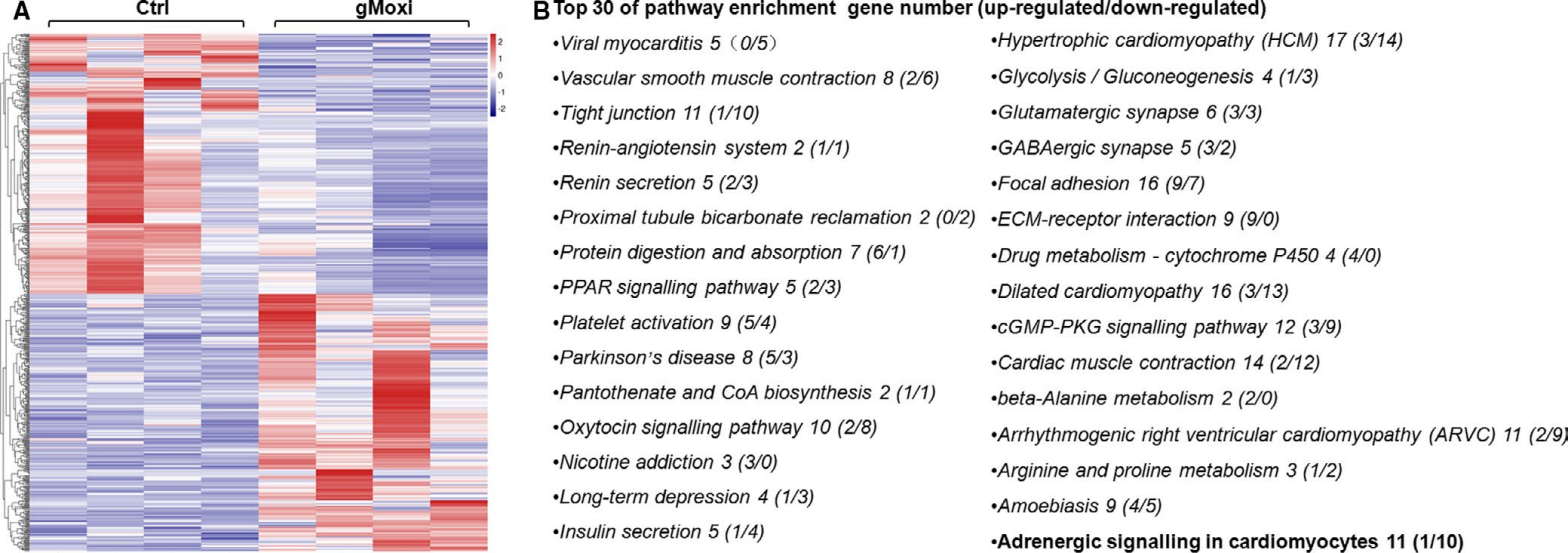
Tumour RNA‐Seq indicates that gMoxi might inhibit adrenergic signalling. Lewis lung cancer (LLC) cells were inoculated on the upper back of C57BL6 mice on day 0, and gMoxi was performed at the acupoint Zusanli (ST36) with three moxa cones every other day from day 1. Mice were killed on day 21, and tumours were isolated and analysed by RNA‐Seq. A, The hot map of DEGs. B, The top 30 KEGG pathways

To further explore the mechanism underlying the gMoxi‐promoted NK cell antitumour immunity, the tumours of mice treated with gMoxi or sham gMoxi were applied to RNA‐Seq. Results showed that a total of 689 genes were differentially expressed. Among these DEGs, 343 genes were up‐regulated, and 346 genes were down‐regulated (Figure 4A). Surprisingly, no clear cancer pathway was enriched by the KEGG analysis. According to the top 30 KEGG pathways, most of the pathways were related to neuroendocrine signalling (Figure 4B). Among these pathways, the adrenergic signalling attracted our attention, because NK cells express more β‐adrenergic receptors than CD8^+^ or CD4^+^ T cells,^12^ and β‐adrenergic signalling plays an important role in NK cells.^13^, ^14^ A total of 11 genes in β‐adrenergic signalling were differentially expressed, including 10 down‐regulated DEGs (Cacna1s, Cacng1, Myl2, Myh7, Myh6, Myl3, Atp1a2, Scn4b, Tnnc1 and Actc1) and one up‐regulated DEG (Tnnt2). This finding indicated that the β‐adrenergic signalling might be inhibited by gMoxi, and enhanced NK cell antitumour immunity might be related to such inhibition.

### 3.5 gMoxi inhibits adrenergic signalling and then promotes NK cell antitumour immunity

**FIGURE 5:**
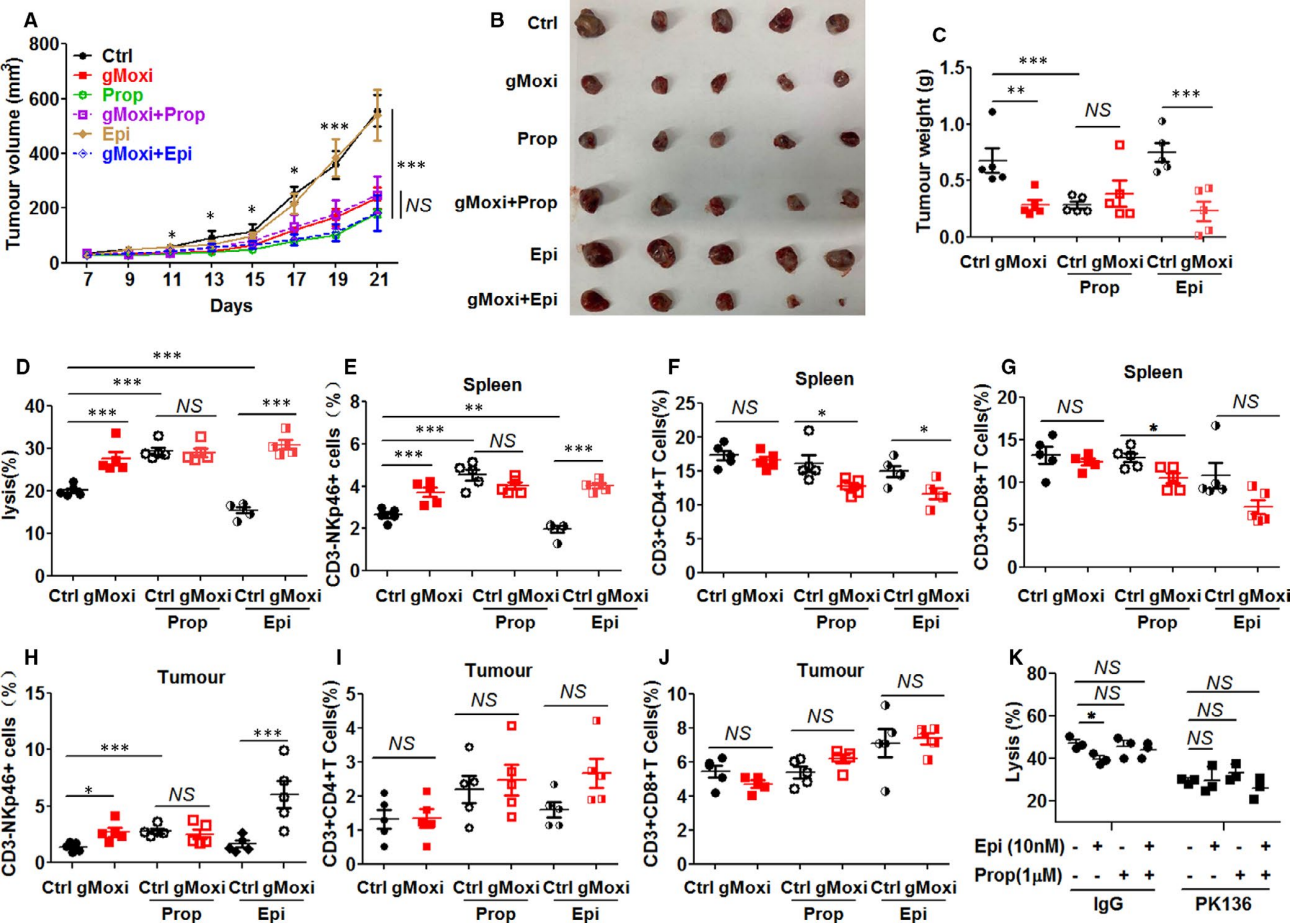
gMoxi‐mediated tumour regression and NK cell promotion depend on adrenergic signalling. Lewis lung cancer (LLC) cells were inoculated on the upper back of C57BL6 mice on day 0, and gMoxi was performed at the acupoint Zusanli (ST36) with three moxa cones every other day from day 1. Propranolol (Prop) in drinking water was given 1 wk before LLC inoculation. Epinephrine (Epi) was daily administered by IP injection from day 1. Mice were killed on day 21, and tumours and spleens were isolated and analysed by flow cytometry. A, Tumour growth curve; B, tumour photograph; C, tumour weight; D, splenocytes were co‐cultured with YAC1 cells at 10:1, and tumour lysis was detected by apoptosis analysis. E, the proportion of CD3^‐^NKp46^+^ NK cells in spleens; F, the proportion of CD3^+^CD4^+^ T cells in spleens; G, the proportion of CD3^+^CD8^+^ T cells in spleens; H, the proportion of CD3^‐^NKp46^+^ NK cells in tumours; I, the proportion of CD3^+^CD4^+^ T cells in tumours; J, the proportion of CD3^+^CD8^+^ T cells in tumours; K, splenocytes were incubated in the present or absent Prop or Epi with or without PK136 antibody for 24 h and then co‐cultured with YAC1 cells at 5:1 for 2.5 h, and tumour lysis was detected by apoptosis analysis. Independent experiments were repeated twice. *P < .05; **P < .01; ***P < .001; NS, non‐statistically significant

To confirm whether gMoxi‐promoted NK cell antitumour immunity by inhibiting adrenergic signalling, propranolol and epinephrine were applied to block and activate adrenergic signalling, respectively. Results showed that propranolol remarkably inhibited LLC tumour growth (Figure 5A‐C) and increased NK cell cytotoxicity (Figure 5D), as well as the proportion in spleens (Figure 5E) and infiltration in tumours(Figure 5H), whereas it did not increase the proportions of CD4^+^ and CD8^+^ T cells in spleens (Figure 5F,G) and infiltration in tumours (Figure 5I,J). After propranolol treatment, gMoxi could not further suppress the LLC tumour growth (Figure 5A‐C) and promote NK cells (Figure 5D,E,H). Epinephrine could decrease NK cell cytotoxicity (Figure 5D) and proportion of NK cells in spleens (Figure 5E). After epinephrine treatment, gMoxi still significantly inhibited LLC tumour growth (Figure 5A‐C) and promoted NK cell cytotoxicity (Figure 5D), proportion (Figure 5E) and infiltration (Figure 5H). Additionally, epinephrine inhibited NK cell–mediated cell killing in vitro, and propranolol could resist this inhibition (Figure 5K). Taken together, these results demonstrated that gMoxi enhanced the antitumour immunity of NK cells by suppressing adrenergic signalling.

## 4 DISCUSSION

Moxibustion has been used to treat cancer‐related symptoms and to alleviate the side effects of surgery, radiotherapy and chemotherapy. However, its antitumour effect on NSCLC remains largely unexplored. In the present study, we found that gMoxi at Zusanli acupoint (ST36) significantly suppressed LLC tumour growth. The mechanistic analysis showed that gMoxi could promote NK cell antitumour immunity by inhibiting adrenergic signalling, resulting in tumour regression.

The efficacy of moxibustion is related to the acupoint site, time and dose. Therefore, those influencing factors were first optimized. We found that gMoxi at Zusanli acupoint (ST36) elicited a better tumour inhibitory effect compared with gMoxi at Mingmen acupoint (DU4) and Guanyuan acupoint (RN4), which are usually used in clinical practice. Furthermore, gMoxi every other day had a better therapeutic effect compared with gMoxi every day, every 2 days or every week. Additionally, gMoxi with three or seven moxa cones had no difference. These results suggested that gMoxi with three moxa cones at the acupoint of Zusanli every other day could elicit an optimized therapeutic efficacy.

The acupoint of Zusanli is an important acupoint closely related to immune modulation, especially to the neuroendocrine‐immune network.^15^ For example, scar‐producing moxibustion by heat stimulating the acupoint Zusanli can effectively decrease the neutrophil‐to‐lymphocyte ratio and improve the life quality in NSCLC patients.^16^ Electroacupuncture at Zusanli promotes macrophage polarization during the fibrotic process by decreasing cytokine IFN‐γ and increasing IL‐4, IL‐13 and IFN‐α.^17^ Bee venom acupuncture at ST36 acupoint can attenuate the development and progression of experimental autoimmune encephalomyelitis (EAE) by up‐regulating regulatory T cells and suppressing T‐helper (Th) 17 and Th1 responses.^18^ In this study, we found that gMoxi at Zusanli could increase the proportion of NK cells in spleens and infiltration in tumours, but had no obvious effect on CD4^+^ and CD8^+^ T cells. Our finding confirmed that Zusanli acupoint was closely related to immune regulation, although the regulatory cells were different. All these studies indicated that the immune response of Zusanli acupoint might be distinct from different treatments in different disease progression.

The effect of adrenergic signalling in NK cells remains contradictory in different studies. Some studies have shown a positive effect. For example, adrenergic signalling promotes NK cell activity to prevent cancer.^19^ Blockade of β‐adrenergic signalling decreases the enriched environment‐induced enhancement of tumour infiltration and cytotoxic activity of NK cells, and attenuates the antitumour effect of enriched environment.^20^ Intrinsic adrenergic signalling is required for optimal adaptive NK cell responses to protect against viral infection.^21^ However, some studies have revealed a negative effect. For example, β‐adrenergic blocker propranolol increases tumour‐infiltrating CD56^+^ NK cells in colorectal cancer.^22^ Sleep deprivation reduces the number and cytotoxicity of NK cells, which can be reversed by β‐adrenergic blocker propranolol.^23^ Norepinephrine inhibits the cytotoxicity of NK92‐MI cells via the β2‐adrenergic signalling pathway.^24^ The different functions of adrenergic signalling in NK cells might be related to the environment and disease progression. In this study, we found that gMoxi could promote NK cell antitumour immunity to suppress tumour growth by inhibiting adrenergic signalling, suggesting that adrenergic signalling negatively regulated NK cell activity and promoted tumour growth in NSCLC. This finding was consistent with the previous studies that adrenergic signalling is a cancer target, and beta‐blockers can improve the clinical outcome of lung cancer patients.^25^ Of course, additional experiments are required to further elucidate the mechanism underlying the inhibitory effects of gMoxi on adrenergic signalling in NSCLC. Taken together, we, for the first time, demonstrated that gMoxi could inhibit adrenergic signalling in NSCLC, suggesting that gMoxi could be used as a promising therapeutic regimen for NSCLC patients.

Natural killer cells are a group of innate lymphoid cells (ILCs) and play a critical role in tumour immune surveillance.^26^ In patients with cancers, NK cells are usually reduced and impaired, and adoptive NK cell immunotherapy has become a promising regimen for cancer treatment. However, the therapeutic efficacy of NK cells is limited in clinical practice owing to tumour escape.^27^ Therefore, it is urgently necessary to improve NK cell antitumour immunity. Although the effect of moxibustion on NK cells has contradictory reports, some studies have demonstrated that moxibustion suppresses the cytotoxicity^28^ and proportion^29^ of NK cells, and others have shown that moxibustion enhances NK cell activity.^30^, ^31^ In the present study, we found that gMoxi could increase the proportion, tumour infiltration and killing activity of NK cells. Our current data suggested that gMoxi had great potential in NK cell–based cancer immunotherapy.

In conclusion, we, for first time, reported that gMoxi at Zusanli could inhibit adrenergic signalling, and then promote NK cell antitumour immunity, leading to tumour regression. Collectively, our findings indicated that gMoxi could be used as an ancient but promising therapeutic regimen for the treatment of NSCLC patients.

## CONFLICT OF INTEREST

The authors have declared that no competing interest exists.

## AUTHOR CONTRIBUTIONS

**Dan Hu:** Investigation (equal); Writing‐review & editing (equal). **Weiming Shen:** Investigation (equal); Writing‐review & editing (equal). **Chenyuan Gong:** Data curation (equal); Formal analysis (equal); Methodology (equal). **Cheng Fang:** Formal analysis (equal); Resources (equal); Validation (equal). **Chao Yao:** Methodology (equal); Software (equal); Validation (equal). **Xiaowen Zhu:** Methodology (supporting); Resources (supporting); Validation (supporting). **Lixin Wang:** Data curation (supporting); Funding acquisition (supporting); Methodology (supporting); Validation (supporting). **Chen Zhao:** Conceptualization (equal); Supervision (equal); Writing‐review & editing (equal). **Shiguo zhu:** Conceptualization (lead); Funding acquisition (equal); Writing‐original draft (lead); Writing‐review & editing (equal).

## Supporting information

Figure S1

## Data Availability

The data that support the findings of this study are available from the corresponding author upon reasonable request.
