# Supplementary material for: Grain‐sized moxibustion promotes NK cell antitumour immunity by inhibiting adrenergic signalling in non–small cell lung cancer
Source: J Cell Mol Med. 2021 Jan 27;25(6):2900–8. doi: 10.1111/jcmm.16320 (PMC7957214; doi:10.1111/jcmm.16320)
Supplement: Supplementary file 1 — Figure S1 [file JCMM-25-2900-s001.docx]

**Figure S1.** The gating strategy for flow cytometry. The lymphocytes were gated, and then NK cells were analyzed by using NKp46 and CD3. CD3^+^ T cells were gated from the lymphocytes and then CD4 and CD8 T cells were analyzed. A, the gating strategy for spleen; B the gating strategy for Tumor.
